# Norepinephrine promotes lung cancer cell progression via PFKFB3-promoted histone lactylation-mediated activation of the NF-κB signaling pathway

**DOI:** 10.1515/biol-2025-1321

**Published:** 2026-05-18

**Authors:** Han Ma, Wenlong Yang, Yiwei Fan, Hui Zou, Xiaoxia Lv

**Affiliations:** Thoracic Surgery Department, North Jiangsu People’s Hospital, Yangzhou City, Jiangsu Province, 225000, China

**Keywords:** norepinephrine, PFKFB3, histone lactylation, lung cancer, NF-κB signaling

## Abstract

This study aims to investigate molecular mechanisms by which norepinephrine (NE) exposure influences lung cancer cell behavior, identify key factors and pathways involved in stress-related signaling in lung cancer cells. NE was used to treat lung cancer cells (H1975) to simulate a stress-related signaling environment. PFKFB3 mRNA expression and phosphorylation levels of epithelial-mesenchymal transition (EMT) markers and key proteins in NF-κB signaling pathway were evaluated. NE significantly enhanced lung cancer cell proliferation, specifically upregulated PFKFB3 mRNA expression, promoted EMT marker (N-cadherin and Vimentin) expression, and increased cell migration capacity. Knockdown of PFKFB3 or inhibition of glycolysis reduced cell viability, lactate production, ATP levels, and expression of glycolysis-related enzymes (LDHA and GLUT1) and EMT markers, while suppressing cell migration. Combined interventions exhibited synergistic inhibitory effects. Mechanistic studies revealed that PFKFB3 knockdown inhibited phosphorylation of NF-κB pathway proteins (IKKβ, IκBα, and p65), blocked pathway activation, and reduced histone lactylation levels, particularly at the H4K12la modification site. These effects were reversed by NE intervention. This study suggests a mechanism whereby NE upregulates PFKFB3, activates glycolysis, promotes lactate production to drive histone lactylation, and activates the NF-κB signaling pathway, which is associated with EMT and cell migration.

## 1 Introduction

Lung cancer, as the malignant tumor with the highest global incidence and mortality rates, has its core pathological basis for poor prognosis rooted in its high metastatic potential and therapeutic resistance [1]. While traditional research has predominantly focused on the driving roles of genetic mutations and epigenetic alterations in tumor progression, a wealth of recent clinical data has unveiled a significant association between chronic stress and adverse outcomes as well as advanced clinical stages in lung cancer patients [2], 3]. Chronic stress may reshape tumor biological behavior through the intricate interplay of neuroendocrine, metabolic, and immune networks. However, the direct effects of norepinephrine on lung cancer cell metabolism and epigenetics require further investigation, necessitating further investigation.

Current research indicates that chronic stress facilitates lung cancer progression through multifaceted mechanisms. At the neuroendocrine level, chronic restraint stress significantly elevates acetylcholine (ACh) levels within the lung adenocarcinoma (LUAD) microenvironment. By activating the α5 nicotinic acetylcholine receptor (α5-nAChR), this process suppresses the expression of the tumor suppressor gene FHIT, thereby augmenting the invasive and migratory capacities of cancer cells [4]. Norepinephrine (NE), via the ADRB2-cAMP-PKA-CREB signaling axis, promotes cancer stemness and chemotherapy resistance [5]. In the realm of immune regulation, chronic stress induces polarization of tumor-associated macrophages (TAMs) toward the M2 phenotype through the HIF1A-AS3/HIF-1α positive feedback loop, fostering an immunosuppressive microenvironment [6]. Stress hormones such as norepinephrine and cortisol can promote lactate production by enhancing the glycolytic capacity (Warburg effect) of tumor cells [7]. Simultaneously, these hormones induce tumor cells to acquire stem-like properties, and anti-apoptotic capacity, thereby fostering a pro-tumorigenic metabolic phenotype [8].

Recent studies show PFKFB3, a key regulator of glycolysis, is pivotal in lung cancer progression [9]. As 6-phosphofructo-2-kinase/fructose-2,6-bisphosphatase 3, PFKFB3 catalyzes the synthesis of F-2,6-BP, thereby activating phosphofructokinase-1 (PFK-1) and propelling glycolysis forward [10]. Clinical sample analyses have revealed significantly upregulated PFKFB3 expression in non-small cell lung cancer (NSCLC), with its expression levels positively correlating with poor prognosis and metastatic potential [11]. Mechanistically, PFKFB3 not only supplies tumor cells with energy substrates by promoting glycolysis but also mediates epigenetic regulation through the metabolic intermediate lactate [12].

Notably, lactate, traditionally regarded as a terminal glycolytic product, has recently been identified as a substrate for histone lactylation [13]. PFKFB3-driven glycolytic reprogramming significantly enhances histone lactylation levels. This type of epigenetic modification is enriched at the promoter regions of key NF-κB signaling pathway genes (such as Ikbkb, Rela, and Relb), activating NF-κB protein nuclear translocation and transcriptional activity, thereby triggering inflammatory-metastatic cascades [14]. The NF-κB pathway, serving as a central regulatory hub within the tumor microenvironment, is directly involved in tumor cell survival and epithelial-mesenchymal transition (EMT) [15], 16]. Its interplay with metabolic reprogramming constitutes a critical nexus in tumor progression. However, the functional association between NF-κB pathway activation and stress-induced metabolic reprogramming as well as histone modifications remains to be fully elucidated.

This study investigates whether NE enhances glycolytic metabolism by activating PFKFB3, and the resultant metabolic intermediate lactate further mediates histone lactylation, potentially activating the NF-κB signaling pathway to promote EMT and cell migration. By exploring metabolic-epigenetic regulatory mechanisms underlying NE signaling, our research identifies potential therapeutic targets for targeted lung cancer interventions.

## 2 Materials and methods

### 2.1 Cells and cell culture

NSCLC cell lines H1975 was sourced from ATCC. NSCLC cells (H1975) were maintained in RPMI-1640 medium (Gibco, Thermo Fisher Scientific) enriched with 10 % fetal bovine serum (FBS, Gibco) and 1 % penicillin-streptomycin (Gibco). BEAS-2B cells were cultured in BEGM BulletKit medium (Lonza) as per the manufacturer’s protocol. All cells were incubated at 37 °C in a 5 % CO_2_ humidified atmosphere. Regular tests confirmed all cell lines were free from mycoplasma contamination.

This study designed three experimental modules to elucidate core mechanisms: 1) To investigate the concentration-dependent effects of norepinephrine (NE), cells were treated with six concentration gradients (0, 0.1, 1, 10, 50, 100 μM) for 48 h. Cell viability was assessed via CCK8 assay, and PFKFB3 expression was analyzed by qRT-PCR. To characterize phenotypic transitions, additional groups including a blank control (Ctrl) and 10 μM NE-treated group were established. EMT marker expression was detected by Western blot, and cell migration capacity was evaluated through wound healing assays. 2) To dissect PFKFB3 functions, five intervention models were constructed: blank control (Ctrl), negative control shRNA (sh-NC), PFKFB3 knockdown (sh-PFKFB3), glycolysis inhibitor 2-DG (10 mM) treatment, and combined intervention (sh-PFKFB3 + 2-DG). 3) To validate epigenetic mechanisms, experimental groups comprised blank control (Ctrl), NE treatment (10 μM), PFKFB3 knockdown, and rescue group (sh-PFKFB3 + 10 μM NE). All experiments were independently triplicated, with data presented as mean ± standard deviation. The shRNAs targeting PFKFB3 (sh-PFKFB3) and a non-targeting control (sh-NC) were designed and synthesized by GenePharma (Shanghai, China). The target sequence for PFKFB3 was CCG​GGC​TGA​TGA​AGA​AGA​TGA​ACA​TTC​AAG​AGA​TG TTCATC TTC​TTC​ATC​AGC​TTT​TTG. The negative control sequence was CCG​GTT​CTC​CGA​ACG​TGT​CAC​GTT​TTG​GAA​T.

### 2.2 Cell viability assay

Cell viability was detected using a Cell Viability Assay Kit (BA00208, Bioss, Beijing, China). The cells in the logarithmic growth phase were inoculated into 96-well plates at 2000 cells per well and incubated under the conditions of 37 °C and 5 % CO_2_ for 4–8 h. After the cells adhered to the wall, they were treated according to the experimental groups, with 6 replicate wells set up in each group. The cells in each group were incubated for 24 h. Two hours before the end of incubation, 10 μL of CCK8 solution was added to each well. After incubation, the OD450 was measured with a microplate reader.

### 2.3 Quantitative RT-PCR

Total RNA was extracted from cells using the Cell/Tissue Total RNA Isolation Kit V2 (RC112, Beyotime, Shanghai, China). RNA concentration and purity were measured using a Nano 600 spectrophotometer (Jiapeng Technology, Shanghai, China). Subsequently, cDNA was synthesized from 1 µg of total RNA using the HiScript III 1st Strand cDNA Synthesis Kit (R312, Beyotime, Shanghai, China). qRT-PCR was performed on a CFX96 Touch real-time PCR detection system (Bio-Rad, USA) using Universal SYBR qPCR Master Mix (MQ101, Beyotime, Shanghai, China). The PFKFB3 mRNA expression level was normalized to the internal reference gene GAPDH. All reactions were performed in triplicate. Relative gene expression was calculated using the 2^−ΔΔCq^ method.

The primer sequences were: PFKFB3 forward, 5′-CCA​CCA​AAA​AGC​CTC​GCA​TC-3′, and reverse, 5′-CTC​CGG​GAG​CCT​TTC​ATG​TT’; GAPDH forward, 5′-AATGGGC AGCCGTTAGGAAA-3′, and reverse, 5′-GCG​CCC​AAT​ACG​ACC​AAA​TC-3′. All primers were from Sangon Biotech.

### 2.4 Lactate production assay

Lactate concentrations in cell culture media were determined using a lactate assay kit (BioVision), following the supplier’s instructions. Cells were cultured in serum-free medium for 24 h, after which the supernatant was collected. Absorbance at 570 nm was measured, and lactate levels were normalized to cell count and expressed as μM per 10^7^ cells.

### 2.5 ATP measurement

Intracellular ATP levels were quantified using an ATP assay kit (Beyotime). Cells were seeded in 6-well plates at a density of 2 × 10^5^ per well and treated with 2-DG (10 mM) or subjected to PFKFB3 knockdown. After 24 h, cells were lysed, and ATP content was measured on a luminometer. Results were normalized to cell number and expressed in nmol ATP per 10^4^ cells.

### 2.6 Western blot analysis

Protein extracts were obtained by lysing cells in RIPA buffer (Thermo Fisher Scientific) supplemented with protease and phosphatase inhibitors (Sigma-Aldrich). Protein concentrations were measured using a BCA protein assay kit (Pierce), and equal amounts (20 μg) were separated by SDS-PAGE, followed by transfer to PVDF membranes (Millipore). Membranes were blocked in 5 % non-fat milk for 1 h, then incubated overnight at 4 °C with primary antibodies against PFKFB3, LDHA, GLUT1, E-cadherin, Snail, *p*-IKKβ, IKKβ, *p*-IκBα, IκBα, p-p65, p65, panKla, anti-H4K12la and GAPDH (all from Cell Signaling Technology). After washing, membranes were incubated with HRP-conjugated secondary antibodies for 1 h. Protein bands were visualized using ECL reagents (Thermo Fisher Scientific) and a ChemiDoc imaging system (Bio-Rad). GAPDH was used as a loading control for all Western blot analyses.

### 2.7 Wound healing assay

Cell motility was examined through a wound healing assay. H1975 cells were plated in 6-well plates to 90 % confluence, and a scratch was created using a sterile 200 μL pipette tip. Cells were washed with PBS and incubated in serum-free medium. Images were taken at 0 and 24 h, and wound closure was quantified as the reduction in wound area relative to the initial area. Each condition was performed in three independent biological replicates, with at least three scratch wounds quantified per replicate.

### 2.8 Statistical analysis

Each experiment was conducted in triplicate. Data are presented as mean ± standard deviation (SD). Statistical comparisons were made using one-way ANOVA followed by Tukey’s post-hoc test for multiple groups, with a significance threshold set at p < 0.05. All analyses were performed with GraphPad Prism (version 9.0).

## 3 Results

### 3.1 Norepinephrine promotes lung cancer cells progression through PFKFB3

**Figure 1:**
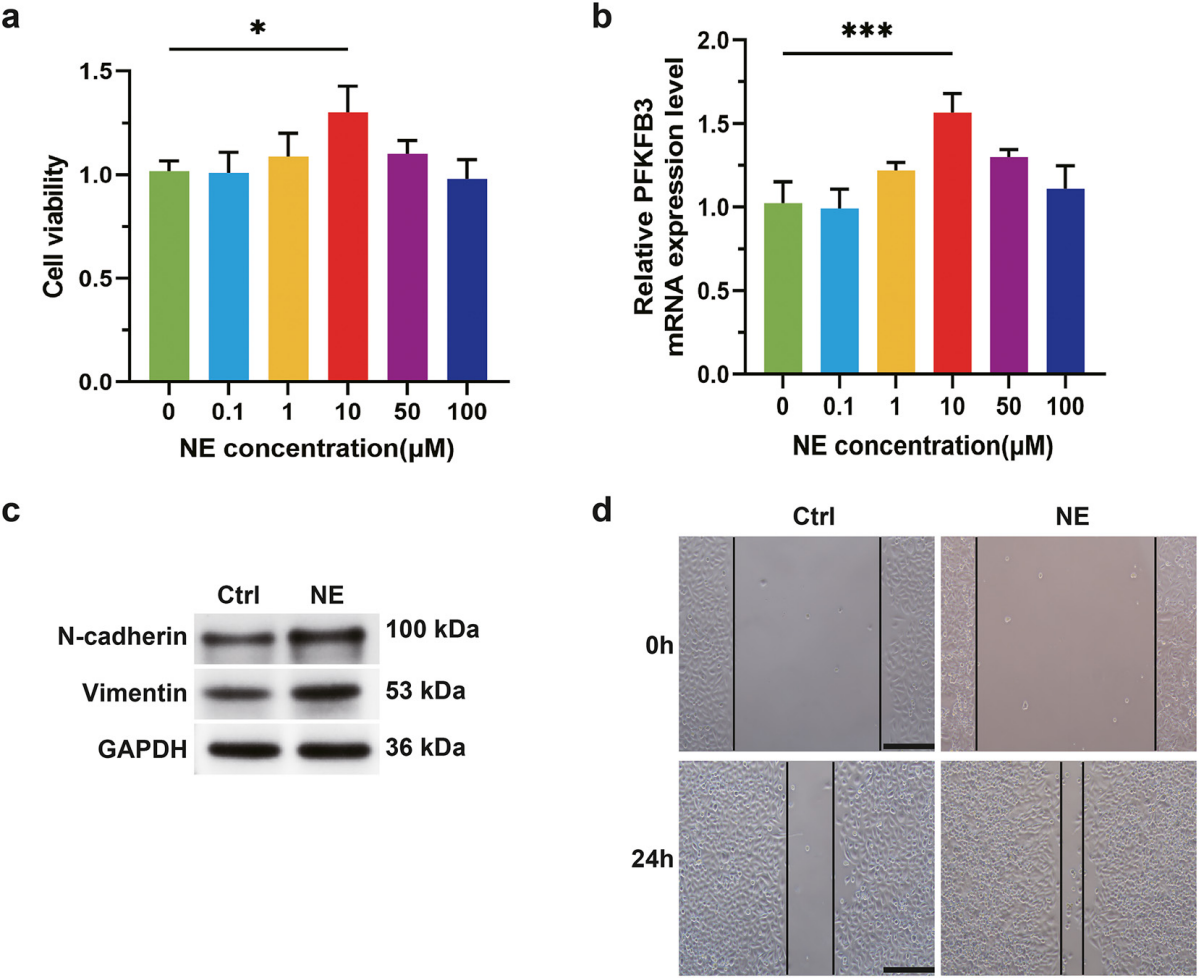
NE promotes PFKFB3 expression and drives lung cancer cells to exhibit proliferative and invasive phenotypes. (a) Cell viability was detected by CCK8. (b) qRT-PCR analysis of the expression levels of PFKFB3. (c) Western blot analysis of the expression levels of EMT markers. (d) The cell migration ability was detected by wound healing assays. The results are expressed as mean ± SEM. *n* = 3 for each group. *p < 0.05, ***p < 0.001. Scale bars: 200 μm.

To elucidate the impact of norepinephrine (NE) exposure on lung cancer progression, this study utilized NE to treat H1975 cells. Concentration gradient screening (0–100 μM) revealed that 10 μM NE significantly enhanced cell proliferation (CCK8 assay) and specifically upregulated PFKFB3 mRNA expression (qRT-PCR), with no notable differences observed at other concentrations (Figure 1a and b). Western blot analysis confirmed that 10 μM NE promoted the expression of epithelial-mesenchymal transition (EMT) markers (N-cadherin and Vimentin) (Figure 1c). Wound healing assays demonstrated that 10 μM NE markedly increased lung cancer cell migration. These findings indicate that NE is associated with lung cancer cell proliferation, EMT progression, and migratory capacity through the upregulation of PFKFB3.

### 3.2 PFKFB3 drives invasive phenotype transformation in lung cancer cells via glycolysis

**Figure 2:**
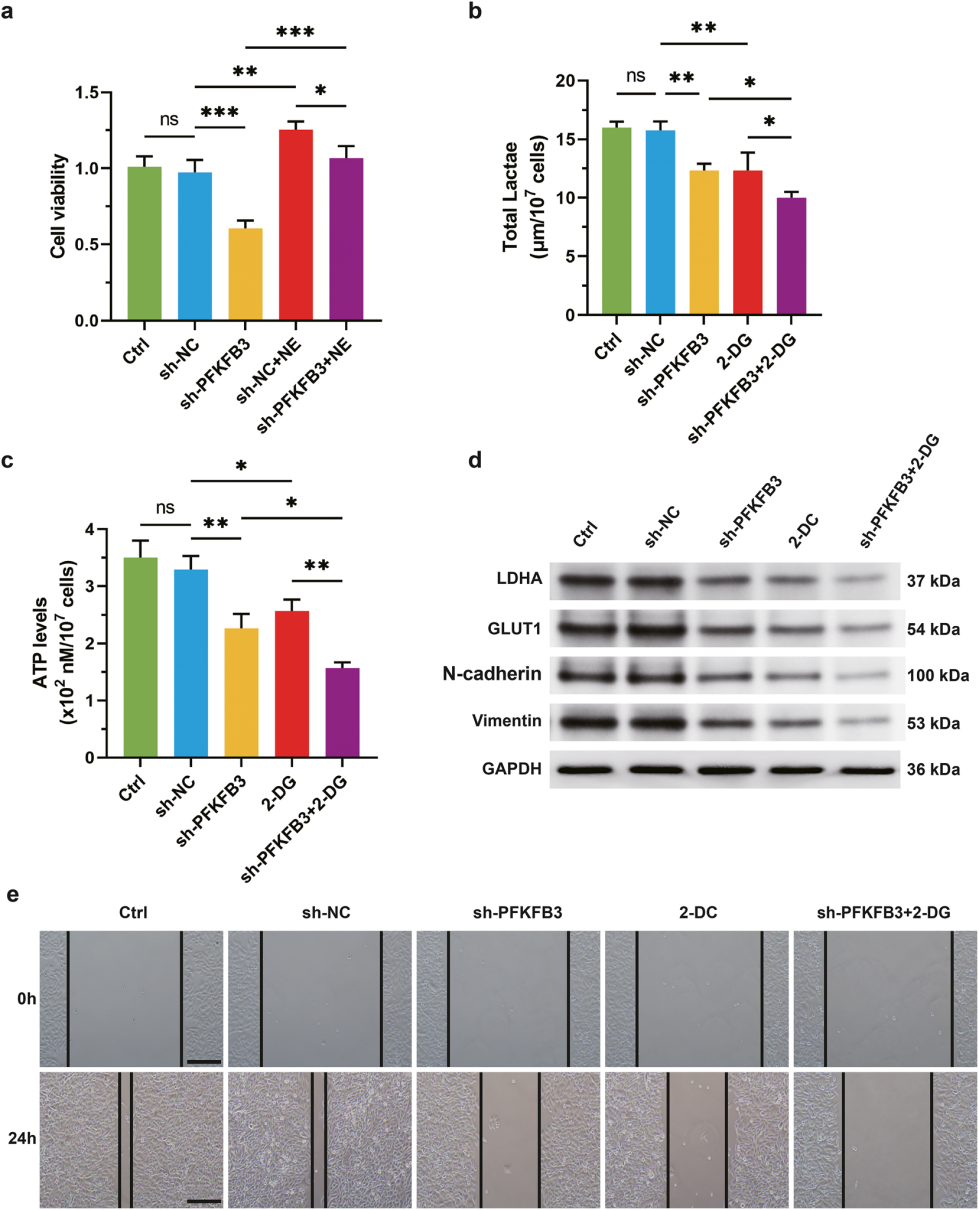
PFKFB3 regulates glycolysis to promote invasive phenotype transformation in lung cancer cells. (a) Cell viability was detected by CCK8. (b) The level of lactate was measured using a lactate assay kit. (c) The level of ATP was measured using an ATP assay kit. (d) Western blot analysis of the expression levels of glycolysis regulators and EMT markers. (e) The cell migration ability was detected by wound healing assays. The results are expressed as mean ± SEM. *n* = 3 for each group. *p < 0.05, **p < 0.05, ***p < 0.001. Scale bars: 200 μm.

To clarify the functional pathway of PFKFB3, metabolic interventions were performed using shRNA-mediated PFKFB3 knockdown (sh-PFKFB3) combined with the glycolysis inhibitor 2-deoxy-D-glucose (2-DG). Results showed that both PFKFB3 depletion and glycolysis inhibition significantly reduced cell viability (Figure 2a), lactate production (Figure 2b), and ATP levels (Figure 2c), confirming PFKFB3 as a critical regulator of cancer cell energy metabolism. Further analysis revealed synchronized downregulation of glycolytic enzymes (LDHA, GLUT1) and EMT markers (N-cadherin, Vimentin) following PFKFB3 knockdown or glycolysis suppression (Figure 2d), suggesting that PFKFB3-driven glycolysis may support metabolic reprogramming for invasive phenotype acquisition. Wound healing assays confirmed that PFKFB3 depletion or glycolysis blockade inhibited cell migration, with synergistic effects observed upon combined intervention (Figure 2e). Collectively, these results suggest that PFKFB3 promotes EMT and cell migration by regulating glycolysis, serving as a key metabolic node in lung cancer progression.

### 3.3 Norepinephrine activates the NF-κB pathway via PFKFB3-mediated histone lactylation to drive lung cancer progression

**Figure 3:**
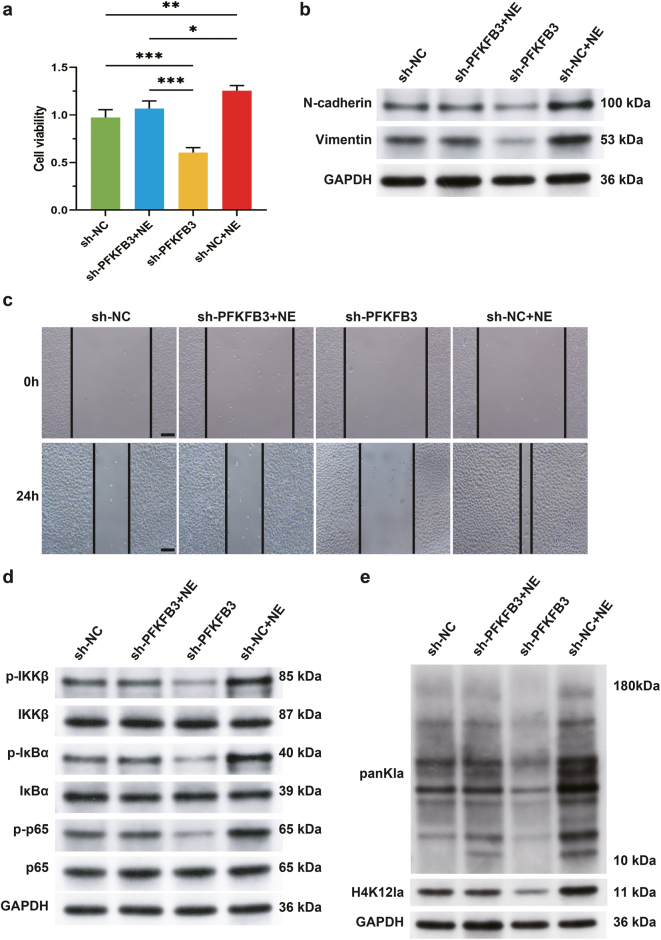
The NE-PFKFB3 axis activates the NF-κB pathway through histone lactylation to drive EMT progression. (a) Cell viability was detected by CCK8. (b) Western blot analysis of the expression levels of EMT markers. (c) The cell migration ability was detected by wound healing assays. (d) Western blot analysis of the activity of NF-κB signaling pathway. (e) Histone lactylation levels were quantified by Western blot using panKla and anti-H4K12la antibody. The results are expressed as mean ± SEM. *n* = 3 for each group. *p < 0.05, ***p < 0.001. Scale bars: 200 μm.

This study further investigates an epigenetic mechanism by which NE promotes malignant phenotypes through PFKFB3. In PFKFB3-knockdown lung cancer cells, reduced cell viability and downregulated EMT markers (N-cadherin, Vimentin) were reversed by exogenous NE supplementation, with wound healing assays confirming restored migratory capacity (Figure 3a–c). Mechanistically, NE was associated with NF-κB pathway activity via PFKFB3: PFKFB3 knockdown decreased phosphorylation of IKKβ, IκBα, and p65, suggesting blocked NF-κB activation, while NE intervention rescued these phosphorylations (Figure 3d). Notably, PFKFB3 depletion suppressed histone lactylation, particularly at the H4K12 lactylation site (H4K12la), which NE restored through PFKFB3 (Figure 3e). H4K12la enrichment at NF-κB target gene promoters has been reported to activate transcription, and NF-κB signaling promotes EMT [14]. Thus, our data suggest that NE may enhance lung cancer metastasis by promoting histone lactylation via PFKFB3, activating the NF-κB pathway, and inducing EMT.

## 4 Discussion

This study investigates norepinephrine (NE) upregulates PFKFB3 expression in lung cancer cells, which is associated with lactate production and histone lactylation, and the activation of the NF-κB signaling pathway, potentially contributing to epithelial-mesenchymal transition (EMT) and migration in lung cancer cells. The findings highlight PFKFB3 and histone lactylation as potential targets for intervention in lung cancer cells exposed to NE.

Chronic stress reshapes the tumor microenvironment to promote cancer malignant progression by activating the sympathetic nervous system to release catecholamine hormones such as NE [8], 17]. This study provides evidence that NE directly upregulates PFKFB3 expression to influence tumor cell metabolic phenotypes, which aligns with previous understanding of chronic stress-induced metabolic regulation via long non-coding RNAs (lncRNAs) [6]. Notably, while the pro-tumor effects of NE mediated by the β2-adrenergic receptor (ADRB2) have been widely confirmed, its direct regulatory role on PFKFB3, a key glycolytic enzyme, has been less explored [18]. This study demonstrates a significant upregulation of PFKFB3 expression and concomitant lactate production in NE-treated lung cancer cells, consistent with the mechanism observed in gastric cancer where PFKFB3 overexpression induces EMT through NF-κB signaling [19]. This suggests a possible conserved regulatory pattern across cancer types. The PFKFB3 inhibitor AZ26 effectively blocks glycolysis and inhibits NF-κB activity, further substantiating its pivotal role as a metabolic-inflammatory signaling hub [20].

Lactate, traditionally regarded as a terminal glycolytic product, introduces a further dimension to tumor biology through its epigenetic regulatory functions [21]. This study confirms that lactate accumulation induced by the NE-PFKFB3 axis drives histone lactylation, particularly a marked elevation in H4K12 lactylation (H4K12la). Analogously, in esophageal cancer, the long non-coding RNA AP001885.4 enhances NF-κB (p65) expression via H3K9 lactylation, while the anti-tumor agent RJA inhibits tumor growth by modulating H3K9la/H3K14la, indicating site-specificity and functional diversity of lactylation modifications [22], 23]. This study links the NE-PFKFB3 signaling axis with histone lactylation, suggesting metabolic stress may regulate tumor progression through epigenetic reprogramming.

The NF-κB pathway, serving as a central node linking chronic inflammation and tumor progression, is subject to multi-dimensional regulation [16], 24]. This study demonstrates that histone lactylation activates the NF-κB signaling pathway, which is in line with reports of NAT10-mediated NF-κB activation via acetylation and suggesting diversity in epigenetic modification modes [25]. Mechanistically, PFKFB3 may orchestrate a positive feedback loop of metabolic-epigenetic-transcriptional regulation by specifically enriching H4K12la at the promoter regions of NF-κB target genes (e.g., Ikbkb, Rela) [14]. This axis upregulates key EMT executors (Snail, Twist) and markers (N-cadherin, Vimentin), and appears to synergize with the PFKFB3–NF-κB-EMT axis in gastric cancer and the TGF-β-NF-κB/NOX4/ROS axis to form a networked regulatory system for tumor invasion and metastasis [26], 27].

The proposed “NE-PFKFB3-lactylation-NF-κB-EMT” axis requires further validation but holds potential translational value: 1) The anti-tumor effects of PFKFB3 inhibitors (e.g., AZ26, PFK-15) in gastric and ovarian cancers provide preclinical support for their further investigation in lung cancer [20], 28]; 2) p300/CBP inhibitors (e.g., C646) reversing H3K18la-mediated NF-κB activation offer potential epigenetic therapeutic strategies [29]; 3) *β*-blockers or ADRB2 antagonists may mitigate pro-tumor effects by blocking stress signal transduction [5], 18]. However, limitations persist: 1) Interventional efficacy needs validation in chronic stress animal models, particularly exploring synergies between PFKFB3 inhibitors and immune checkpoint blockades; 2) The synergistic effects of corticosteroids and other stress hormones require investigation to construct multi-hormonal regulatory networks; 3) Interactions between lactylation and other epigenetic modifications (e.g., acetylation, methylation), such as competitive or cooperative mechanisms between H3K9la and H3K9ac in gene regulation, warrant further exploration.

In conclusion, this study systematically investigates the molecular response of NE-treated lung cancer progression through the metabolic-epigenetic axis. It contributes to the understanding of tumor cell responses to NE and provides a basis for further research towards precision therapeutic strategies. This study is limited to *in vitro* experiments using a single NSCLC cell line (H1975) under acute NE exposure. The involvement of adrenergic receptors (e.g., ADRB2) was not directly tested, and future studies should include receptor blockade or knockdown experiments to confirm signaling specificity. Additionally, the translational relevance of these findings requires validation *in vivo* and in clinical samples.

## 5 Conclusions

In summary, our *in vitro* study demonstrates that NE upregulates PFKFB3, enhances glycolysis and histone lactylation, and activates NF-κB signaling in H1975 lung cancer cells, which is associated with EMT and migration. These observations suggest that PFKFB3 is not only a core regulator of glycolysis but also may serve as linking metabolic reprogramming with epigenetic regulation, forming a potential mechanism for promoting lung cancer cell phenotypes through a metabolic-epigenetic-inflammatory signaling axis. This work identifies several candidate targets along the PFKFB3-histone lactylation-NF-κB pathway for further exploration in the context of stress-related signaling in lung cancer.
